# The role of human hunters and natural predators in shaping the selection of behavioural types in male wild turkeys

**DOI:** 10.1098/rsos.240788

**Published:** 2024-11-06

**Authors:** Nick A. Gulotta, Patrick H. Wightman, Bret A. Collier, Michael J. Chamberlain

**Affiliations:** ^1^ Warnell School of Forestry and Natural Resources, University of Georgia, Athens, GA, USA; ^2^ School of Renewable Natural Resources, Louisiana State University, Baton Rouge, LA 70803, USA

**Keywords:** animal personality, harvest-induced selection, among-individual variation, natural selection, hunting, behavioural type

## Abstract

The expression of behaviour can vary both among (i.e. behavioural types (BTs)) and within individuals (i.e. plasticity), and investigating causes and consequences of variation has garnered significant attention. Conversely, studies quantifying harvest-induced selection (HIS) on behavioural traits have received significantly less attention, and work investigating HIS and natural selection simultaneously is rare. We studied sources of variation in three movement traits that represented risk-taking and one trait that represented exploration in male eastern wild turkeys (*Meleagris gallopavo silvestris*). We used data from 109 males in two hunted populations located in Georgia and South Carolina, USA. We assessed how both hunters and natural predators simultaneously influenced the selection of male turkey BTs. We found significant among-individual variation in all movement traits and adjustments in risk-taking and exploration relative to whether hunting was occurring. We observed that predators selected against similar BTs across both populations, whereas hunters selected for different BTs across populations. We also demonstrated that significant HIS acts on risk-taking behaviours in both populations, which could render wild turkeys more difficult to harvest if these traits are indeed heritable.

## 1. Introduction

Phenotypic traits expressed in wild populations experience dynamic selective pressures that lead to shifts in trait optima over time [1,2]. Modifications in phenotypic traits are shaped by the environment and agents of natural selection, such as predators [3,4]. Predators play a significant role in driving phenotypic changes in prey species by non-randomly selecting for certain phenotypes that are easier to exploit [5–7]. Similarly, evidence is accumulating that humans are another major evolutionary force acting on phenotypes that often outpaces selection from natural drivers [8–11]. Human-induced changes in phenotypes are usually the most conspicuous when humans act as the primary predator by harvesting wild populations [12–15]. Harvesting can exert significant selective pressures by decreasing survival rates [16–18], altering population dynamics [19,20] and selectively targeting specific phenotypes in a process known as harvest-induced selection (HIS) [12,21–26].

An aspect of HIS that has received less attention in the literature involves the non-random selection of behavioural traits [12]. Non-random selection of behaviours stemming from harvest can be attributed to among-individual differences in behaviour (i.e. behavioural types (BTs)), wherein certain behavioural characteristics of individuals influence the likelihood of being harvested [12,21–23,26]. For instance, research on the movement behaviour of elk (*Cervus elaphus*) has demonstrated that individuals who express riskier behaviours, such as a preference for open areas, and those with greater exploratory tendencies are more likely to be harvested [21]. In terrestrial systems, HIS on behavioural traits has received limited attention collectively [12,21,22,26,27], but studies conducted underscore the harvest of individuals exhibiting a fast phenotype, characterized by greater risk-taking, activity and exploration [12,21–23,26]. Furthermore, most research on HIS has been conducted within the context of fisheries [15,25,28]. This body of work, including a recent review by Leclerc *et al.* [12], provided a detailed framework for exploring hypotheses and predictions about the influence of HIS on behavioural traits. For example, it is hypothesized that in hunted populations, traits that increase detectability to hunters, such as occurring closer to open areas, displaying boldness or greater activity levels, could make animals more susceptible to harvest [12]. Likewise, several studies within the fisheries literature have concurrently explored the impact of HIS and natural selection on behavioural traits, revealing divergent preferences for behavioural phenotypes between predators and humans [28,29]. Despite the insights revealed by examining HIS and natural selection simultaneously [27,28], there remains a notable absence of research on the combined effects of HIS and natural selection on the behaviour of terrestrial game species.

Male wild turkeys (*Meleagris gallopavo silvestris*) provide a unique opportunity to simultaneously assess HIS and natural selection, stemming from the fact that male wild turkeys are primarily harvested during the reproductive period, which is when natural predation for males is also heightened [16,17,30–32]. Wild turkeys are a non-migratory upland game bird that use a polygamous–promiscuous mating system in which females select the most attractive males for copulation [30]. To attract and secure mating opportunities, males engage in courtship behaviours such as vocalizations and elaborate displays [30–35], which are inherently risky behaviours that can attract predators. Males that engage in courtship behaviours will often use open landcover for displaying and vocalizing [36–38] as open areas facilitate increased visual perception and reduced sound attenuation, increasing the opportunity for attracting potential receptive females at greater distances [39–41].

Previous studies have found that male wild turkeys alter movements during the hunting season and increase movements prior to and during peak incubation to search for receptive females [33,42–44]. This period of increased competition and mate searching coincides with increased mortality risk from hunters and predators, with hunting accounting for most male mortalities [16,17,45]. Earlier work on the movement behaviour of wild turkey hunters demonstrated that hunters do not move far from hunting access points, and primarily use secondary roads which are comprised of extensive edges that contain early successional plant communities used by wild turkeys [46,47]. Moreover, edge landcover functions as travel corridors and foraging areas for significant wild turkey predators like bobcats (*Lynx rufus*) and coyotes (*Canis latrans*), with the latter exhibiting increased interactions with male wild turkeys [42,48]. As such, behaviours involving the use of edge landcover, open landcover, closer proximity to hunting access points and greater exploratory tendencies pose significant mortality risks, as these actions increase the likelihood of encountering both predators and hunters [42,46–48]. Nevertheless, despite the mortality risks linked to these behaviours, our understanding of how risk-taking and exploratory behaviours affect the survival of male wild turkeys remains limited.

Wild turkey abundance, productivity and harvest across broad areas of the species range have been steadily declining [49,50], whereas predators of wild turkeys have exhibited increases in abundance [51]. Despite numerous studies exploring survival rates, harvest rates and movement characteristics of male wild turkeys [16,17,42–44], there is still a limited understanding of the effects of HIS and natural selection on behavioural phenotypes. Moreover, although recent research has explored how hunting pressure affects the movement behaviour of male wild turkeys [42], there is a notable absence of studies comparing differences in movement behaviour across multiple locations subjected to hunting pressure. Our objectives were to investigate (i) whether wild turkeys are being harvested based on their BT (i.e. average phenotypic expression); (ii) whether wild turkeys with specific BTs were being non-randomly removed by predators; (iii) whether hunters and predators selected similar or divergent BTs; (iv) if hunters and predators were selecting for similar BTs across two hunted populations; and (v) whether behavioural responses to hunting pressure were similar across both hunted populations. We hypothesized that hunters and predators would both non-randomly select certain behavioural phenotypes that were easier to exploit. Similarly, we hypothesized that both predators and hunters would select similar BTs across both populations. We predicted that in both populations, hunters and predators would select BTs characterized by greater levels of risk-taking and exploration. Our logic for this prediction was that greater expressions of risk-taking and exploration would lead to increased interactions with both hunters and predators, ultimately negatively impacting male turkey survival. Finally, we hypothesized that male wild turkeys would adjust their behaviour in response to hunting pressure, and we predicted that adjustments of movement behaviour would be in a similar direction for each behavioural trait across both populations.

## 2. Methods

### 2.1. Study area

During 2017–2023, we conducted research on two wildlife management areas (WMAs) and surrounding private lands located in the Piedmont region of Georgia, USA. Cedar Creek WMA (CCWMA) was approximately 16 187 ha, B.F. Grant WMA (BFGWMA) was approximately 4613 ha and both were managed in cooperation with the Georgia Department of Natural Resources—Wildlife Resources Division (GADNR). The CCWMA was owned by the United States Forest Service, whereas BFGWMA was owned by the Warnell School of Forestry and Natural Resources at the University of Georgia. For analysis, we considered males in BFGWMA and CCWMA as belonging to a single population (hereafter referred to as Georgia) since the two WMAs were separated by 4.55 km and individuals from these sites clustered genetically into a single population [52]. During 2014–2018, we also conducted work on the Webb WMA Complex (hereafter referred to as South Carolina), which was approximately 10 483 ha and consisted of three contiguous WMAs (Hamiliton Ridge WMA, Palachacola WMA and Webb WMA) located near the Savannah River in Garnett, South Carolina, USA, and managed by the South Carolina Department of Natural Resources (SCDNR). In Georgia, a total of 1316 ha of open areas were available, featuring large agricultural fields and pastures (>10 ha), alongside smaller openings (<2 ha) that featured planted plots designed to attract wildlife. In contrast, in South Carolina only 180 ha of open areas were present, and openings primarily consisted of small food plots (<2 ha) established to attract wildlife. For further information on the study sites and hunting seasons at each site, see electronic supplementary material, S1.

### 2.2. Data collection

We captured wild turkeys from January to March annually using rocket nets baited with cracked corn, and upon capture each individual was marked with an aluminium leg band, and fitted with a remotely downloadable backpack-style GPS-VHF-UHF transmitter (Biotrack Ltd, Wareham, Dorest, UK and e-obs GmbH, Gruenwald, Germany [53]). We aged wild turkeys based on the presence of barring on the 9th and 10th primary feathers, and characterized individuals as either adults or juveniles [30]. Capture, handling and marking procedures were approved by the Institutional Animal Care and Use Committee at the University of Georgia (A2019 01-025 R2) and Louisiana State University (A2014‐13).

Due to battery constraints of the GPS transmitters, data were only collected for approximately 1 year on each individual; thus we used data collected from 1 March until 31 July for each year of the study. We used data during this time period because it spanned the breeding season and hunting season in their entirety, which are when male wild turkeys face the greatest levels of mortality risk [16]. All GPS transmitters were programmed to record one location nightly (23:58:58), birds captured in Georgia recorded locations hourly from 05.00 to 20.00 hours, whereas in South Carolina locations were recorded every 30 minutes in accordance with project objectives [54]. We located each individual and downloaded GPS data using handheld antennas and receivers once a month and monitored survival 3–5 times a week. At mortality sites, we used field signs to identify predator guilds responsible for killing wild turkeys, such as avian predators like great horned owls (*Bubo virginianus*) and red-tailed hawks (*Buteo jamaicensis*) and mammalian predators like bobcats (*Lynx rufus*) and coyotes (*Canis latrans*). However, we combined all predator-related deaths due to insufficient sample sizes for analysing survival rates specific to predator guilds. We also sent wild turkeys that died with no visible trauma to the Southeastern Cooperative Wildlife Disease Study (SCWDS) at the University of Georgia, where they performed necropsies and X-rays. We assigned hunting mortalities to those recovered and reported directly by hunters or when we determined the cause of death resulted from crippling loss under the following three circumstances: a transmittered bird that was with another harvested transmittered bird and was found dead within 48  hours of the harvest and the carcass was indicative of crippling; a transmittered bird was known to be shot at by a hunter and visually wounded based on a conversation with the hunter, which likely resulted in mortality; and a transmitter bird was recovered dead during the wild turkey hunting season with no apparent signs of predation, and during necropsy we found evidence of gunshot pellets throughout the bird’s carcass. We considered instances where the transmitter harness had been cut and discarded to be illegal kills and we included such mortalities as harvest. If a wild turkey was found dead or determined a hunter harvest, we truncated data to include only GPS points when the individual was alive. We cleaned raw GPS data and excluded any GPS locations that did not have adequate satellite coverage and had dilution of precision (DOP) values > 7 [55].

### 2.3. Behavioural data

Juvenile male wild turkeys were rarely harvested in our study populations and contemporary evidence suggests that adults are primarily targeted by hunters [16]. Therefore, we used GPS data from adult male wild turkeys and focused on three metrics that we believed represented risk-taking (i.e. distance to edge landcover, distance to open landcover and distance to hunter access) and one metric that represented exploratory tendencies (average hourly speed). To calculate our risk-taking metrics, we first generated individual raster layers for forested (pine, hardwood, shrub and mix pine/hardwoods) and open landcover types using 30 m × 30 m raster layers from the United States Department of Agriculture (USDA), National Agricultural Statistical Service for each year of the study [56]. The USDA cropland data layers update annually and can account for changes in forest cover from active timber harvest, which occurred on both study sites. Next, we obtained road data from GADNR and SCDNR for roads inside the WMAs and used USGS Tiger/Line data (Topologically Integrated Geographic Encoding and Referencing) for roads outside the WMAs that traversed private lands. We characterized secondary roads as either logging roads and/or unpaved gravel roads that did not allow vehicular access, whereas primary roads were paved/gravelled and vehicle access was not limited. We created an edge landcover feature by identifying all areas of the landscape where the forested raster layers were adjacent to open raster layers for either road type. We then used ArcGIS 10.5.1 (Environmental System Research Institute, Inc., Redlands, CA, USA) to calculate a distance-based raster for edge and open landcover on both study sites. We completed the remainder of the analyses using R v. 4.3.1 [57]. We used package raster [58] and sf [59] to calculate the distance from GPS points to nearest open and edge landcover types, which allowed us to obtain continuous behavioural measurements rather than using classification or categorical approaches [60].

We recognize that at our Georgia study site, certain areas were unreachable to hunters from primary roads due to private property blocking access to public land, whereas in South Carolina, no private properties restricted access to public land. However, at both study sites, seasonal road closures occur to reduce vehicle travel rendering portions of both study sites more difficult to access. Therefore, we also computed the closest distance to hunter access points (see above methods) where the general public could park a vehicle and access public hunting areas, assuming that areas farther from hunter access points would experience less hunting activity [42,46,47]. For each distance to metric (i.e. distance to open landcover, edge landcover, hunter access points), we calculated the average daily distance for each male turkey and used these metrics for further analysis. Finally, to compute our metric for exploratory behaviour (i.e. average hourly speed) we calculated the total distance travelled within a given day by each male turkey using package adehabitatLT [61] and sp [62]. We then divided that value by 15 (i.e. hours within a day that the GPS transmitter collected data) for each individual to calculate their average hourly speed.

### 2.4. Statistical analysis

We conducted our analysis in three steps, first by constructing four separate univariate models, each incorporating one of the four behavioural traits, using the brms package [63]. We used the function *scale()* to standardize each response variable prior to analysis to assist with model convergence. Subsequently, we categorized GPS data into three separate time frames: the pre-hunt stage (from March to the beginning of the hunting season), the hunt stage (during the hunting season) and the post-hunt stage (from the day after the hunting season ended until 31 July). We then fitted random intercepts for individual wild turkeys, and fixed effects of hunting stage (pre-hunt, hunt, post-hunt), study population (Georgia, South Carolina) and an interaction between hunting stage and study population. We felt justified in including a fixed effect for hunting stage since prior research has demonstrated that male wild turkeys adjust their movement behaviour before, during and after the hunting season [42]. Likewise, we included an interaction between hunting stage and study population to statistically control and test differences in behavioural traits between study populations. Next, to assess the existence of among-individual variation (i.e. BTs) in each behavioural trait, we calculated repeatability, which measures the amount of phenotypic variance attributed to individual variation [64,65]. We considered any behavioural trait with a repeatability value of *r* > 0.3 as showing moderate repeatability [64]. To calculate repeatability, we extracted the posterior distribution for the among-individual variance and residual variance, and we used the following formula [64]:


(2.1) $$repeatability=\;\frac{{Vind}_{0}}{{Vind}_{0}+\;V_{e0}},$$

where Vind_0_ was the among-individual variance and *V_e_*_0_ was the residual within-individual variance.

For all univariate models, we performed 15 300 iterations of each model with a burn in of 300, a thinning interval of 15, and we used two Markov chains per model. We used the default prior in the brms package [63], and assessed convergence using *R*-hat values and visually inspected trace and density plots. We considered *R*-hat values < 1.1 to show adequate model convergence. For all models, we report the median (*β*) of the posterior distribution, 95% credible intervals (CrI) and probability of direction (PD) (i.e. proportion of the posterior distribution that is in the direction of the estimated effect) to evaluate the support for each effect [66,67]. We describe effects that did not have CrI overlapping zero as showing strong support for an effect, and we considered CrI centred around zero (with an equal distribution on both sides of zero) with an effect estimate close to zero and a PD <89% as strong support for no effect. We considered CrI that overlapped zero with a PD ≥89% as showing moderate support for an effect [66,67].

### 2.5. Survival analysis

We first attempted to fit an additional multivariate model that estimated the among-individual correlation between behaviour (trait with repeated measurements) and survival (single measure of survival per individual), but the model would not converge. Therefore, we used a generalized linear model (GLM) with a binary response variable for survival as a function of best linear unbiased predictors (BLUPs) for each behaviour extracted from univariate models [68–71]. This approach allowed us to incorporate the uncertainty associated with using single values of BLUPs by iterating through the entire distribution of BLUP values for each individual [68], and resulted in 2000 unique GLMs for each behavioural trait that we used to assess the effects of behaviours on survival. We used the 2000 estimated effects to calculate a total effect size for all 2000 iterations [69–71]. We report the median effect size for all 2000 iterations and 95% CrI for the relationship between each behavioural trait and survival and used the same interpretation for significance of effects as noted above.

To ease interpretation, we refitted risk-taking models (average daily distance to hunter access, average daily distance to open landcover, average daily distance to edge landcover) and multiplied each trait by −1 prior to analysis. This allowed us to interpret a positive log odds ratio as more risky individuals having greater survival, and shyer individuals having lower survival, whereas we interpreted a negative log odds ratio as more risky individuals having lower survival and shyer individuals having greater survival. Next, we subset the data to include individuals that either survived or were harvested in the year they were monitored (Georgia—*n* = 36 survived, *n* = 13 harvested; South Carolina—*n* = 29 survived, *n* = 14 harvested). We then fitted survival as a binary response as a function of BLUP values for each behavioural trait separately using the approach stated above, which allowed us to directly estimate if greater levels of risk-taking and exploratory behaviour were unintentionally selected for by hunters. Likewise, we used the same approach to assess if BTs were selected for by predators by only including data on individuals that survived or were predated (Georgia—*n* = 36 survived, *n* = 10 predated; South Carolina—*n* = 29 survived, *n* = 7 predated).

## 3. Results

### 3.1. Univariate models assessing behavioural types

From Georgia, we used data on 59 adult males, resulting in 5185 daily values with an average of 88 repeated measures per individual for each behavioural trait (minimum = 7, maximum = 153). For South Carolina, we used data on 50 adult males, resulting in 4557 daily values with an average of 91 repeated measures per individual for each behavioural trait (minimum = 13, maximum = 155). Collectively, there were 3284 behavioural observations during the pre-hunt stage, 2772 observations during the hunting stage and 3686 in the post-hunt stage (see electronic supplementary material, tables S1 and S2, for more detailed summary statistics on observations per site and per individual).

**Figure 1.**
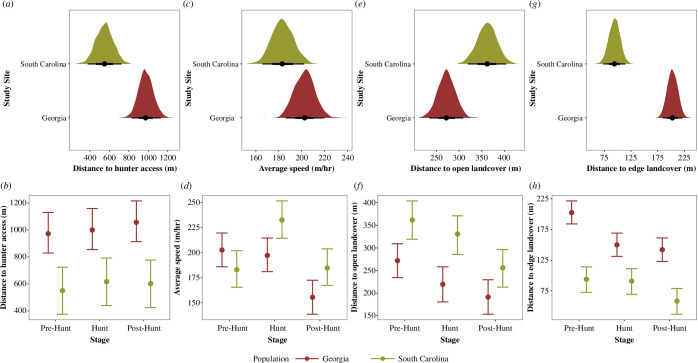
Univariate results for the effect of population and hunting stage interacted with population for adult male wild turkeys at South Carolina (*n* = 50; data spans 2014–2018) and Georgia (*n* = 59; data spans 2017–2023). (*a*,*c*,*e*,*g*) The posterior distribution, 95% CrI and posterior median are presented for the effect of population for each trait. (*b*,*d*,*f*,*h*) Posterior median and 95% Crl are displayed for the interaction between population and hunting stage. (*a*,*b*) Distance to hunter access. (*c,d*) Average hourly speed. (*e,f*) Distance to open landcover. (*g,h)* Distance to edge landcover.

We found for each behavioural trait moderate to high repeatability, which confirmed the existence of consistent among-individual differences in risk-taking and exploration (table 1). Furthermore, we found that on average, individuals from South Carolina displayed riskier behaviour and occurred closer to edge landcover and hunter access points (distance to edge—*β* = −0.88, 95% CrI = −1.14, −0.64, PD = 100%; distance to hunter access—*β* = −0.56, 95% CrI = −0.87, −0.25, PD = 100%), whereas in Georgia we found a greater investment in only one risk-taking measure, distance to open landcover (*β* = −0.42, 95% CrI = −0.68, −0.14, PD = 99.95%). We also detected adjustments in behaviour in response to hunting stage that varied in strength and direction between the two study populations (table 1; figure 1). For instance, in South Carolina individuals had increased exploratory tendencies during the hunting season compared to the pre-hunting stage (*β* = 0.50, 95% CrI = 0.44, 0.57, PD = 100%), whereas in Georgia we failed to detect a difference in exploration between the pre-hunt and hunting stage since ROPE (region of practical equivalence) values were >98% which demonstrated no effect (*β* = −0.05, 95% CrI = −0.10, −0.00, PD = 97.25%, ROPE = 98.58%). However, we found that exploration did decrease after the hunting stage in both study populations (South Carolina—*β* = −0.49, 95% CrI = −0.55, −0.43, PD = 100%; Georgia—*β* = −0.43, 95% CrI = −0.48, −0.38, PD = 100%). In addition, for both study populations we found a similar response to distance to open landcover during the hunting stage, whereas individuals from both study populations increased risk-taking as the hunting stages progressed and occurred closer to open landcover (table 1; figure 1). We also found that in both study populations, individuals increased risk-taking and occurred closer to edge landcover after the hunting stage (table 1; figure 1). However, we did not detect any differences in distances to edge landcover between the pre-hunt and hunt stage in South Carolina (*β* = 0.02, 95% CrI = −0.03, 0.08, PD = 81.39%), but in Georgia we did detect an increase in risk-taking during this same period (*β* = 0.43, 95% CrI = 0.38, 0.48, PD = 100%). Finally, in South Carolina, we failed to detect differences in distance to hunter access across all hunting stages (table 1; figure 1). In Georgia, we did detect a decrease in risk-taking with individuals occurring farther from hunter access after the hunting stage compared to the pre-hunt stage (*β* = −0.11, 95% CrI = −0.15, −0.08, PD = 100%, ROPE = 22.11%).

**Table 1.** Univariate analyses of sources of variation in risk-taking behaviours and exploration for adult male wild turkeys in Georgia (*n* = 59) and South Carolina (*n* = 50). The study period covered 2014–2018 in South Carolina and 2017–2023 in Georgia. We scaled all behaviours prior to analysis, and for each model fitted the behavioural trait as the response variable and fixed effects of hunting stage, population and an interaction between hunting stage and population. We interpreted lower distance values as greater levels of risk-taking, and greater values of speed as fast exploration. Results for the repeatability (*r*) for each behavioural trait are presented. Repeatability quantifies the amount of variance that is attributed to individual differences in behaviour. We report beta estimates (*β*) as posterior medians, 95% CrI and PD. Any estimate that overlapped zero but had a PD ≥89% was interpreted as a significant effect.

fixed effects	distance to hunter access^a^		average speed^b^		distance to open landcover^c^		distance to edge landcover^d^	
	*β* (95% CI)	PD (%)	*β* (95% CI)	PD (%)	*β* (95% CI)	PD (%)	*β* (95% CI)	PD (%)
**intercept^e^**	0.20 (−0.01, 0.41)	97.05	0.05 (−0.12, 0.22)	69.10	−0.02 (−0.19, 0.16)	56.80	0.57 (0.42, 0.74)	100
**hunting stage**								
**hunting**	0.04 (0.00, 0.07)	97.80	−0.05 (−0.11, 0.00)	96.80	−0.25 (−0.30, −0.20)	100	−0.43 (−0.48, −0.38)	100
**post-hunt**	0.11 (0.08, 0.15)	100	−0.48 (−0.54, −0.42)	100	−0.38 (−0.43, −0.33)	100	−0.50 (−0.55, −0.45)	100
**hunting stage: population**								
**hunt:South Carolina**	0.05 (0.00, 0.11)	97.80	0.56 (0.48, 0.65)	100	0.10 (0.02, 0.17)	99.60	0.41 (0.33, 0.48)	100
**post hunt:South Carolina**	−0.04 (−0.10, 0.01)	94.10	0.50 (0.41, 0.58)	100	−0.12 (−0.20, −0.05)	99.95	0.21 (0.13, 0.28)	100
**population**								
**South Carolina**	−0.56 (−0.87, −0.25)	100	−0.19 (−0.44, 0.05)	93.65	0.43 (0.15, 0.69)	99.90	−0.88 (−1.14, −0.64)	100
**random effects**	***σ* (95% CI)**		***σ* (95% CI)**		***σ* (95% CI)**		***σ* (95% CI)**	
**individual ID**	0.67 (0.49, 0.86)		0.44 (0.32, 0.56)		0.47 (0.35, 0.59)		0.35 (0.26, 0.45)	
**residual**	0.22 (0.22, 0.23)		0.58 (0.56, 0.60)		0.44 (0.42, 0.45)		0.45 (0.43, 0.46)	
**repeatability**	***r* (95% CI)**		***r* (95% CI)**		***r* (95% CI)**		***r* (95% CI)**	
**individual ID**	0.74 (0.69, 0.79)		0.43 (0.36, 0.50)		0.51 (0.45, 0.58)		0.44 (0.38, 0.51)	

^a^ Distance to hunter access.

^b^ Average speed.

^c^ Distance to open landcover.

^d^ Distance to edge landcover is transformed prior to analysis (see §2 for details).

^e^ Intercept is for reference categories pre-hunting stage and Georgia.

### 3.2. Survival analysis

**Figure 2.**
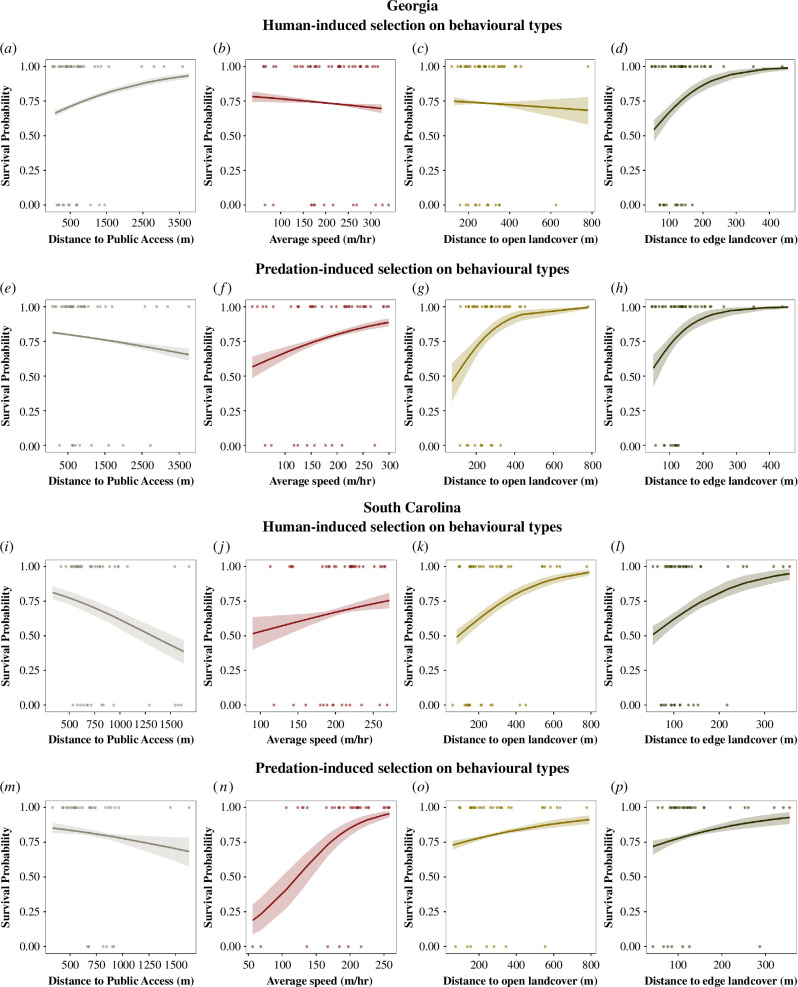
Findings from the survival analysis of adult male wild turkeys in Georgia (*a–h*) and South Carolina (*i–p*) during 2017–2023 and 2014–2018, respectively. Each dot represents an individual’s mean BLUP value extracted from univariate models (*a–d, n* = 49 individuals; *e–h, n* = 46 individuals; *i–l, n* = 43 individuals; *m*–*p, n* = 36 individuals) and binary survival outcome (1—survived, 0—dead). The posterior median (solid line) and 95% CrI (shaded ribbon) are plotted for the predicted probability of survival. (*a–d,i–l*) Results of human selection of BTs (individual average phenotypic expression). (*e–h,m–p*) Results of predator selection of BTs.

For each behavioural trait, we analysed the influence of HIS on BTs. In Georgia, we found that hunters harvested individuals exhibiting more risky BTs, such as occurring closer to hunter access points (log odds ratio = −0.40, 95% CrI = −0.48, −0.33, PD = 100%) and closer to edge landcover (log odds ratio = −1.40, 95% CrI = −1.88, −1.00, PD = 100%). We did not find support that hunters harvested individuals that occurred closer to open landcover types (log odds ratio = −0.10, 95% CrI = −0.09, 0.29, PD = 85.10%), but hunters did harvest individuals that were fast explorers (log odds ratio = −0.16, 95% CrI = −0.28, −0.03, PD = 99.35%). In contrast, in South Carolina, we generally found the opposite pattern, as hunters harvested individuals exhibiting less risky BTs such as occurring farther away from hunter access points (log odds ratio = 1.10, 95% CrI = 0.86, 1.37, PD = 100%), and being slow explorers (log odds ratio = 0.58, 95% CrI = 0.15, 0.98, PD = 99.40%). However, like individuals in Georgia (figure 2; table 2), we did find that hunters were more likely to harvest individuals that occurred closer to edge landcover types (log odds ratio = −1.17, 95% CrI = −1.65,−0.79, PD = 100%).

**Table 2.** Results of survival analysis for each behaviour and mortality source for adult male wild turkeys in South Carolina and Georgia. The study period covered 2014–2018 in South Carolina and 2017–2023 in Georgia. Survival (1—survived, 0—dead) was fitted as the response and BLUP values for each individual were fitted as a fixed effect using data on adult males from Georgia (*n* = 36 survived, *n* = 13 harvested, *n* = 10 predated) and South Carolina (*n* = 29 survived, *n* = 14 harvested, *n* = 7 predated). Beta estimates (*β*) from the analysis are presented as log-likelihoods with 95% CrI. A positive log-likelihood was interpreted as higher expression of a behavioural trait equals higher survival, and lower expression of a trait equals lower survival. While a negative log-likelihood was interpreted as a higher expression of a behavioural trait and lower survival, and lower expression of a trait equals higher survival. We report posterior medians, 95% CrI and PD. Any estimate that overlapped zero but had a PD ≥89% was interpreted as a significant effect.

			distance to hunter access		average speed		distance to open landcover		distance to edge landcover	
fixed effect	study population	mortality source	*β* (95% CrI)	PD (%)	*β* (95% CrI)	PD (%)	*β* (95% CrI)	PD (%)	*β* (95% CrI)	PD (%)
**BLUP**	Georgia	human	−0.40 (−0.48, −0.33)	100	−0.16 (−0.28, −0.03)	99.35	0.10 (−0.09, 0.29)	85.10	−1.40 (−1.88, −1.00)	100
	South Carolina	human	1.10 (0.86, 1.37)	100	0.58 (0.15, 0.98)	99.40	−0.96 (−1.15, −0.78)	100	−1.17 (−1.65, −0.79)	100
	Georgia	predator	0.17 (0.12, 0.22)	100	0.67 (0.47, 0.89)	100	−1.75 (−2.50, 1.09)	100	−1.79 (−2.70, −1.08)	100
	South Carolina	predator	0.57 (0.10, 0.98)	99.00	2.23 (1.78, 2.74)	100	−0.40 (−0.58, −0.25)	100	−0.63 (−1.02, −0.35)	100

We found that for both populations, natural selection acted in a similar direction for all four behavioural traits but varied in strength among traits (table 2; figure 2). Individuals that expressed riskier BTs, such as occurring closer to open landcover (Georgia—log odds ratio = −1.75, 95% CrI = −2.50, −1.09, PD = 100%; South Carolina—log odds ratio = −0.40, 95% CrI = −0.58, −0.25, PD = 100%) and edge landcover (Georgia—log odds ratio = −1.79, 95% CrI = −2.70, −1.08, PD = 100%; South Carolina—log odds ratio = −0.63, 95% CrI = −1.02, −0.35, PD = 100%), were more likely to suffer mortality from predators. Predators selected for slow explorers (Georgia—log odds ratio = 0.67, 95% CrI = 0.47, 0.89, PD = 100%; South Carolina—log odds ratio = 2.23, 95% CrI = 1.78, 2.74, PD = 100%) and individuals that occurred farther from hunter access points (table 2; figure 2).

## 4. Discussion

We demonstrated significant repeatable variation across all three risk-taking behaviours, as well as our single measure of exploratory behaviour, which is similar to other studies using individual movement data to partition phenotypic variance into among- and within-individual components [22,72,73]. In addition, we observed site-specific differences in behavioural trait repeatability (see electronic supplementary material, table S3) and found that all four traits adjusted in response to hunting stages, with the strength and direction of these adjustments varying across populations. Similar findings of behavioural adjustments in response to hunting pressure have been documented in numerous studies of other game species [74–76], including wild turkeys [42,43,77].

We found that in both study populations, male wild turkeys exhibited increased risk-taking behaviours by moving closer to open landcover as the hunting stages advanced, an observation reported previously [42]. Prior research on resource selection of female wild turkeys has demonstrated that females selected open landcover throughout the duration of the reproductive period [78]. Selection of open landcover by females likely influences male wild turkeys in both of our study populations, because selecting open landcover would enhance their chances to encounter reproductively active and receptive females, which is consistent with findings from other species that use a mating system similar to wild turkeys [79,80]. Our findings also indicate that male wild turkeys modify their proximity to edge landcover based on the hunting stages, as we observed that males from both populations exhibited increased risk-taking from the pre-hunt to the post-hunt stage. We offer three plausible, non-mutually exclusive, explanations for these behaviours. Males might be using edge landcover as corridors to more efficiently locate reproductively active females. Alternatively, considering that females often choose nesting sites near roads and edge landcover [81,82], males could be attracted to these areas to monitor the nesting status of females. Finally, previous resource selection studies noted that edge landcover was important for female wild turkeys by offering quality foraging and escape cover [82], a relationship that likely extends to males as well.

Our findings lend support to a hypothesis outlined in Leclerc *et al*. [12], wherein individuals occurring closer to open landcover are more likely to be detected and harvested by hunters. However, we only found support for this hypothesis in South Carolina, whereas in Georgia, we found no evidence of HIS influencing risk-taking. Given that openings are important to wild turkeys [36–38], and that wild turkeys persistently seek out open landcover even when it is scarce [83], our observations in South Carolina suggest a unique scenario. Males in South Carolina increased risk-taking during the hunting season by occurring closer to open land cover, but open areas were limited to smaller food plots (<2 ha) meant to attract wildlife. We speculate that this behaviour led males to become more predictable and conspicuous to wild turkey hunters, which negatively impacted male survival. Conversely, in Georgia, the landscape contained large agricultural fields and pastures (>10 ha) in conjunction with smaller openings that were designated as food plots. As such, it was not surprising that we failed to observe HIS for males in Georgia, since a diverse set of openings were available for male wild turkeys to use, which likely made their use of openings less predictable and conspicuous to hunters.

Our research demonstrates that edge landcover influences the propensity of male wild turkeys being harvested. In both populations, individuals exhibiting riskier behaviour (i.e. occurring closer to edge landcover) had lower survival rates. Lower survival rates for individuals occurring closer to edge landcover was not unexpected, considering that previous research on hunter movements using GPS data indicated that hunters primarily used secondary roads to locate wild turkeys [46,47], which are surrounded by substantial edge landcover. Nevertheless, our study shows significant HIS affecting risk-taking behaviours (Georgia—proximity to edge landcover, distance to hunter access; South Carolina—distance to open landcover, distance to edge landcover) in both study populations. Given that contemporary research on movements of wild turkey hunters has shown similar patterns of space use and hunting tactics across various sites and landscapes, such as using open and edge landcover types to locate wild turkeys [46,47,84], hunters may need to adjust their hunting strategies if wild turkeys alter their behaviour due to the increased mortality risks associated with these landcover types. Additionally, if risk-taking behaviour is found to be heritable and results in evolutionary change (i.e. changes in mean population values of these traits), hunters may need to further adapt [85,86]. We encourage future research to explore the heritability of risk-taking behaviours, especially considering that previous studies on roe deer (*Capreolus capreolus*) have demonstrated relatively high heritability in GPS-based movement behaviours using comparable metrics, such as average speed, distance to roads and use of open landcover [87].

Our research contributes to an increasing body of evidence suggesting that harvest non-randomly targets specific behavioural traits [21–23,25,26]. For male wild turkeys in Georgia, we found that hunters selected to harvest wild turkeys that were fast explorers and took more risks, especially those closer to hunter access points. Conversely, in South Carolina, we observed a different trend: individuals who were faster explorers and riskier (i.e. closer proximity to hunter access points) experienced higher survival rates. Prior research on terrestrial game species has exclusively examined HIS within a single study site [21–23,26]. Conversely, our findings highlight the complexity of HIS, demonstrating that it can differ in magnitude and direction across different study populations. To our knowledge, our study is the first to reveal divergent selection pressures on behavioural traits imposed by hunters across two different populations subjected to similar methods of harvest. Consistent with previous research, our study also confirms that predators selectively target specific BTs [5,7]. Notably, we observed that predators exploited the same BTs across both populations. For example, we found that being closer to edge and open landcover adversely affected survival across both populations. We surmise that male wild turkeys using edge and open landcover types might equate to higher encounter rates with key predators [42], leading to a decrease in survival. Additionally, our data indicate that faster explorers generally had better survival rates than their slower counterparts. Thus, individuals with faster, more superficial exploratory behaviours might not only have more effective anti-predator escape tactics but could also acquire greater spatial memory of potential refuges where predation pressure is lower, consistent with work on a hunted population of brown bears (*Ursus arctos*) where more exploratory individuals had greater survival rates compared to less mobile ones [26].

Similar to suggestions outlined in Leclerc *et al*. [12], we recommend that future investigations into HIS focus on selection regimes at the individual site level, as overlooking this aspect might lead to incorrect management actions intended to mitigate selection exerted on certain phenotypes through harvesting. We acknowledge that such monitoring programmes require continuous funding and a considerable amount of time. Nonetheless, quantifying selection pressures from both hunting and natural selection can be advantageous for devising harvesting regimes [13]. Several previous studies have highlighted that shifting harvest regimes to mimic natural selection could contribute to enhancing the behavioural diversity within a population [12,28,29,88,89]. However, empirical studies that evaluate trait distributions following the manipulation of harvesting regimes to mimic natural selection are currently lacking, although simulation-based studies have shown that such harvest regimes can amplify directional changes in trait frequencies [90]. Therefore, until research demonstrates that directional selection is not amplified under harvest, we recommend adopting conservative harvesting practices that avoid bias towards specific behavioural phenotypes. Preserving behavioural diversity is essential for enhancing a population’s resilience to environmental changes and emerging pathogens. Simultaneously, harvest regimes that are unbiased towards specific behavioural phenotypes could improve hunter satisfaction by preventing the elimination of phenotypes that are easier to harvest, thus maintaining a continuum of individuals that vary in their degree of harvestability within the population [12]. Moreover, additional studies should aim to measure the ecological consequences of HIS, specifically regarding its effects on social hierarchy and sexual selection. These aspects have been shown to be influenced by HIS in other contexts and warrant detailed investigation [91,92]. Additionally, we recognize that differences in available landcover types across study sites may also contribute to the observed patterns. Therefore, we recommend that future studies investigate whether variations in landcover availability influence common movement metrics. Finally, we recommend more studies in terrestrial game species that properly partition phenotypic variance into its among- and within-individual components. An individual-based approach is crucial for comprehending the complex dynamics of HIS and natural selection, ultimately aiding in the refinement of theoretical frameworks.

## Data Availability

Data and relevant code for this research work are stored in Dryad [93] and GitHub [94] and have been archived within the Zenodo repository [95]. Electronic supplementary material is available online [96].
